# The Mitochondrial Peptidase Pitrilysin Degrades Islet Amyloid Polypeptide in Beta-Cells

**DOI:** 10.1371/journal.pone.0133263

**Published:** 2015-07-20

**Authors:** Hanjun Guan, K. Martin Chow, Eunsuk Song, Nirmal Verma, Florin Despa, Louis B. Hersh

**Affiliations:** 1 Department of Molecular and Cellular Biochemistry, University of Kentucky, Biomedical Biological Sciences Research Building, 741 South Limestone St., Lexington, KY, 40536–0509, United States of America; 2 Department of Molecular and Biomedical Pharmacology, University of Kentucky, 459 Wethington Bldg., 800 Rose St., Lexington, KY, 40536–0200, United States of America; University of Akron, UNITED STATES

## Abstract

Amyloid formation and mitochondrial dysfunction are characteristics of type 2 diabetes. The major peptide constituent of the amyloid deposits in type 2 diabetes is islet amyloid polypeptide (IAPP). In this study, we found that pitrilysin, a zinc metallopeptidase of the inverzincin family, degrades monomeric, but not oligomeric, islet amyloid polypeptide *in vitro*. In insulinoma cells when pitrilysin expression was decreased to 5% of normal levels, there was a 60% increase in islet amyloid polypeptide-induced apoptosis. In contrast, overexpression of pitrilysin protects insulinoma cells from human islet amyloid polypeptide-induced apoptosis. Since pitrilysin is a mitochondrial protein, we used immunofluorescence staining of pancreases from human IAPP transgenic mice and Western blot analysis of IAPP in isolated mitochondria from insulinoma cells to provide evidence for a putative intramitochondrial pool of IAPP. These results suggest that pitrilysin regulates islet amyloid polypeptide in beta cells and suggest the presence of an intramitochondrial pool of islet amyloid polypeptide involved in beta-cell apoptosis.

## Introduction

Type 2 diabetes is characterized by a loss of beta cell mass and function, along with the deposition of amyloid in pancreatic islets [1–4]. The main constituent of islet amyloid is islet amyloid polypeptide (IAPP, amylin). Human IAPP (hIAPP), but not rodent IAPP (rIAPP), forms oligomers and fibrils leading to the amyloid deposits associated with type 2 diabetes [3]. What causes beta-cell death when hIAPP aggregates is still debated [3, 5], however evidence suggests hIAPP oligomers as the toxic species. hIAPP oligomers are found within beta-cells in hIAPP transgenic mice [6, 7] where they disrupt membranes [8]. As a consequence of endoplasmic reticulum membrane disruption, hIAPP oligomers induce apoptosis through endoplasmic reticulumstress [9, 10].

Similarities exist between the relationships of hIAPP to type 2 diabetes and human amyloid beta peptide (hAβ) to Alzheimer’s disease [11]. In both disorders toxic peptide oligomers are formed and mitochondrial dysfunction plays an important role [12–15]. In Alzheimer's disease, mitochondria are a direct site of Aβ accumulation [16, 17] with Aβ transported into mitochondria through translocases [18]. In type 2 diabetes mitochondrial dysfunction is associated with insulin resistance in peripheral tissues [13, 14] and with pancreatic beta-cell dysfunction [14, 15]. Exogenous hIAPP induces mitochondrial dysfunction in INS-1E cells [19], however the mechanism is not fully understood.

The mitochondrial peptidase, pitrilysin, cleaves mitochondrial Aβ [20], however the ability of pitrilysin to cleave IAPP has not been investigated until now. We have studied the cleavage of IAPP by pitrilysin and provide evidence that pitrilysin is likely involved in the regulation of IAPP *in vivo*.

## Materials and Methods

### Animals

hIAPP transgenic mice (FVB/N-Tg(Ins2-IAPP)RHFSoel/J) (Jackson laboratory, Bar Harbor, Maine, USA) accumulate amyloid in the pancreas and develops hyperglycemia [6, 21]. Pancreas sections from homozygous 3-month-old male mice were used in this study.

Human islets were purchased from Prodo Laboratories (Irvine, CA, USA).

### Materials

Cell culture reagents were from Invitrogen (Carlsbad, CA, USA). hIAPP was from Anaspec (Fremont, CA, USA). Adenoviruses (Adv) expressing green fluorescent protein (GFP), prepro-rIAPP-GFP, and prepro-hIAPP-GFP were generously provided by Dr. Christopher Rhodes (University of Chicago, Chicago, IL, USA) and expanded as described [22]. For immunoblotting, immunocytochemistry and immunoprecipitation the following antibodies were used: rabbit anti-amyloid oligomer A11, (Millipore, Billerica, MA, USA), mouse anti-hIAPP (Abcam, Cambridge, MA, USA), rabbit anti-hIAPP (Bachem, San Carols, CA, USA), monoclonal anti-hIAPP antibody (E-5, Santa Cruz Biotechnology, Santa Cruz, USA), rabbit polyclonal anti-human/rat IAPP (Bachem-Peninsula Laboratories, San Carlos, CA, USA), mouse anti-insulin (Cell Signaling, Danvers, MA, USA), rabbit anti-pitrilysin (Pierce, Rockford, IL, USA), mouse anti-VDAC1 (Millipore, Billerica, MA, USA), rabbit anti-cleaved caspase-3 (Cell Signaling Technology, Danvers, MA, USA), mouse anti-β-actin (EMD Chemicals, Gibbstown, NJ, USA), and mouse anti-Flag (M2, Sigma). Rabbit anti-mitochondrial malate dehydrogenase (mMDH) antibody was generously provided by Dr. Arnold W. Strauss (Vanderbilt University, Nashville, TN, USA). Rabbit anti-cytosolic thiolase antibody was generously provided by Dr. Sidney W. Whiteheart (University of Kentucky, Lexington, KY, USA). Rabbit anti-human pitrilysin [23] and rabbit anti-insulin-degrading enzyme (IDE) [24] were as described.

### Production and purification of recombinant pitrilysin

Pitrilysin was produced in Sf9 cells (ATCC, Manassas, VA, USA) and purified as described. [23]. The purity of recombinant pitrilysin is greater than 97% as judged from Coomassie blue stained SDS-PAGE gels using NIH ImageJ software [25] (**S1 Fig**).

### In vitro cleavage of hIAPP by pitrilysin

Monomeric hIAPP (20 μM) was incubated with 40 nM pitrilysin in 50 mM Tris/HCl buffer (pH 7.4) containing 200 mM NaCl at 37°C for various times and analyzed by reverse phase HPLC [22, 23]. hIAPP and its cleavage products were collected manually and identified by mass spectrometry.

### In vitro cleavage of oligomeric hIAPP by pitrilysin

hIAPP was incubated at 37°C for 1 hr. with gentle shaking to induce hIAPP oligomerization. To produce low molecular weight oligomers, hIAPP oligomerization was initiated in saline solution at 37°C using 50 μM recombinant hIAPP and then incubated with myocytes for 2 hours at room temperature [26]. Similarly cardiac myocytes from hIAPP expressing rats (HIP rats) contain amylin oligomers and thus both were used. Pitrilysin was added to oligomers and incubated for 1 hour at 37°C. The reaction was then subjected to SDS-PAGE and Western blotting with A11 antisera for large oligomer or with mouse anti-hIAPP for detection of monomers and small oligomers. Adult cardiac myocytes were isolated from wild-type Sprague-Dawley rats and from HIP rats as described [27].

### Cell culture

The rat insulinoma cell line INS 832/13 was kindly provided by Dr. Christopher Newgard (Duke University, Durham, NC, USA) and cultured as described [28, 29].

### Immunocytochemistry and confocal microscopy

INS 832/13 cells were plated on coverslips and transfected with pcDNA3.1-pitrilysin-flag [23] or pCMV6-XL5-prepro-hIAPP (Origene, Rockville, MD, USA) using lipofectamine 2000 (Invitrogen). Cells were fixed 48 hrs. post transfection with 4% paraformadehyde and used for immunofluorescence staining. Pancreas samples from hIAPP transgenic mice were fixed with 10% formalin, embedded in paraffin, and sectioned at 5μm thickness. Sections were deparaffinized followed by antigen unmasking with heat treatment. Cells or sections were permeabilized with 0.1% Triton X-100, blocked with 10% normal goat serum and then incubated with the appropriate antibody overnight at 4°C. Antibodies used were mouse anti-hIAPP (1:100), rabbit anti-mitochondrial malate dehydrogenase (1:200), mouse anti-insulin (1:1000), mouse anti-Flag (M2) (1:200), mouse anti-VDAC1 (1:100), or rabbit anti-pitrilysin (1:100). Cells or sections were then incubated with fluorescein isothiocyanate and/or Cy3-conjugated goat IgG (1:100; Jackson ImmunoResearch, West Grove, PA, USA) and mounted in Vectashield (Vector Laboratories, Burlingame, CA, USA). Confocal microscopy images were taken with a Nikon A1 resonance scanning microscope (Nikon Instruments Inc., Melville, NY, USA) using a 40× or 60× oil immersion objective and a 4× optical zoom. Colocalization of hIAPP with a mitochondrial marker was measured as described previously [30]. Briefly, the 2 channels of hIAPP or insulin and mitochondrial MDH double staining were transformed into 8-bit grayscale images and thresholded. The grayscale images for hIAPP/insulin and mMDH were then merged, and the total pixels of hIAPP or insulin (X), mMDH (Y), and the merged images (Z) were determined using Image J software. The percentage of hIAPP/Insulin that colocalized with mMDH is defined as (X + Y–Z)/Y × 100.

### Knockdown of pitrilysin and IDE production in INS 832/13 cells

GIPZ lentiviral shRNAmir vectors against rat pitrilysin (V3LMM_494519), rat IDE (V3LHS_312518) and a non-silencing control (RHS4346) were from Open Biosystems (Huntsville, AL, USA). These and other lentiviruses were produced according to the manufacturer’s instructions. For selection of INS 832/13 stable cells in which the level of pitrilysin or IDE was knocked-down, cells were transduced with the appropriate lentivirus and then selected with 10 μg/ml puromycin. Knockdown of pitrilysin or IDE was confirmed by Western blot analysis.

### Overexpression of pitrilysin

pcDNA3.1 vectors containing cDNAs of human pitrilysin (PITRM) [23] or its inactive E107Q mutant [20] (PITRMx) tagged with a flag sequence were digested with BamHI/XhoI and subcloned into the lentiviral vector pLenti6/V5 (Invitrogen). For selection of INS 832/13 stable cells overexpressing pitrilysin or inactive pitrilysin, cells were transduced with the appropriate lentivirus and then selected with 10 μg/ml blasticidin. Overexpression was confirmed by Western blot analysis. Empty lentiviral vector was used as a control.

### Cytotoxicity Studies

INS 832/13 cells were transduced with adenovirus expressing prepro-hIAPP-GFP at a multiplicity of infection (MOI) of 100 and cultured for 48 hours. Adenovirus expressing prepro-rIAPP-GFP and GFP alone transduced at the same MOI were used as controls. Apoptosis was determined by the amount of activated caspase-3 [10, 22, 31].

### Isolation of mitochondria

Mitochondria were isolated from INS 832/13 cells by differential centrifugation followed by Percoll purification [32].

### Protease protection assay

Purified mitochondria were resuspended in mitochondria resuspension buffer (250 mM mannitol, 5 mM HEPES, pH 7.4 and 0.5 mM EGTA) at 0.4 mg total protein/ml and incubated for 30 min at 37°C with 10 μg/ml trypsin in the presence or absence of 0.1% Triton X-100. Trypsin digestion was terminated by addition of 50 μg/ml trypsin inhibitor. SDS-PAGE loading buffer was then added to the reaction followed by boiling and analysis by SDS-PAGE and immunoblotting as detailed below.

### Western blot analysis

Protein concentration was determined using the BCA protein assay kit (Thermo Scientific, Rockford, IL, USA). Cell lysates were separated on 4–12% Bis-Tris NuPAGE gels (Invitrogen) and transferred to polyvinylidine difluoride membranes. For Western blotting of IAPP membranes were boiled in PBS for 5 min before blocking. The following primary antibodies were used: rabbit anti-amyloid oligomer (1:6,000), rabbit anti-cleaved caspase-3 (1:3,000), mouse anti-β-actin (1:10,000), rabbit anti-IDE (1:5000), rabbit anti-pitrilysin (1:5000), goat anti-calnexin (1:300), rabbit anti-cytosolic thiolase (1:6,000), rabbit anti-mitochondrial malate dehydrogenase (1:12,000), or rabbit anti-hIAPP (1:3,000). Following incubation with the appropriate primary antibody membranes were incubated with the corresponding horseradish peroxidase-conjugated secondary antibody (Zymed Laboratories, San Francisco, CA, USA) followed by SuperSignal West Pico Chemiluminescent Substrate (Thermo Scientific) and then detected by luminescence. In some cases membranes were reprobed after stripping with stripping buffer (GM Biosciences, Rockville, MD, USA).

### Statistical analysis

Data are expressed as the mean ± SEM and compared using the Student’s t-test. Values are considered to be statistically significant with a p<0.05.

### Ethics Statement

Animal protocols were approved by the University of Kentucky Animal Care and Use Committee and performed in accordance with University guidelines.

## Results

### Cleavage of IAPP by pitrilysin

**Fig 1:**
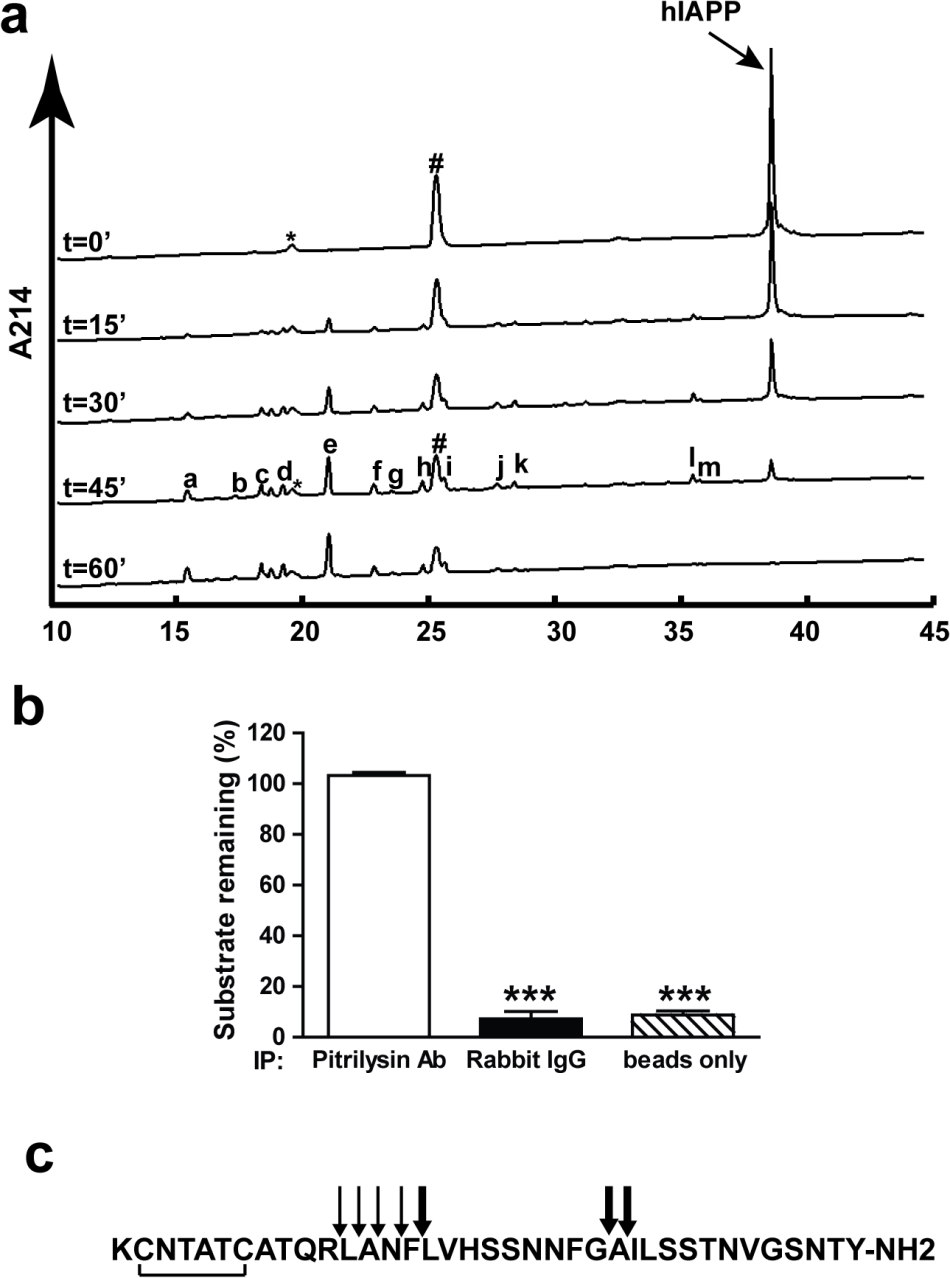
Degradation of monomeric hIAPP by pitrilysin. hIAPP (20 μM) was incubated with 40 nM pitrilysin at 37°C with the reaction followed by HPLC. Peaks were collected and identified by mass spectral analysis. **a.** HPLC chromatograms of hIAPP degradation by pitrilysin at the indicated times. * substrate impurity; # buffer contamination; and a-m hIAPP cleavage products by pitrilysin. **b.** Immunodepletion of pitrilysin reduces hIAPP degradation activity. Purified pitrilysin was incubated with a polyclonal rabbit anti-pitrilysin antibody, normal rabbit IgG, or PBS followed by incubation with protein A/G-Sepharose beads. The beads were spun down and the supernatants incubated with hIAPP. The hIAPP remaining was detected by HPLC as described in Methods. **c.** Schematic of the cleavage sites on hIAPP. Thick arrows represent initial cleavages on hIAPP by pitrilysin and thin arrows represent secondary cleavages.

As noted in the introduction, the zinc metallopeptidase pitrilysin has been shown to cleave amyloid beta peptides [20]. Since IAPP (amylin) and amyloid beta peptides share many common features we tested whether IAPP is a substrate for pitrilysin. To demonstrate the cleavage of monomeric IAPP by pitrilysin, recombinant human pitrilysin was incubated with human IAPP (hIAPP) and the reaction followed by HPLC, **Fig 1A**. To confirm that the observed degradation of hIAPP was mediated by pitrilysin, the purified enzyme was incubated with rabbit anti-pitrilysin antibody followed by protein A/G Sepharose to immunodeplete the enzyme from solution. As shown in **Fig 1B**, hIAPP degradation was abolished when pitrilysin was immunodepleted with rabbit anti-pitrilysin antibody, but not with normal rabbit IgG or a vehicle control, confirming that hIAPP degradation is indeed catalyzed by pitrilysin. Pitrilysin also degrades monomeric rat IAPP (rIAPP), similar to monomeric hIAPP (data not shown).

**Fig 2:**
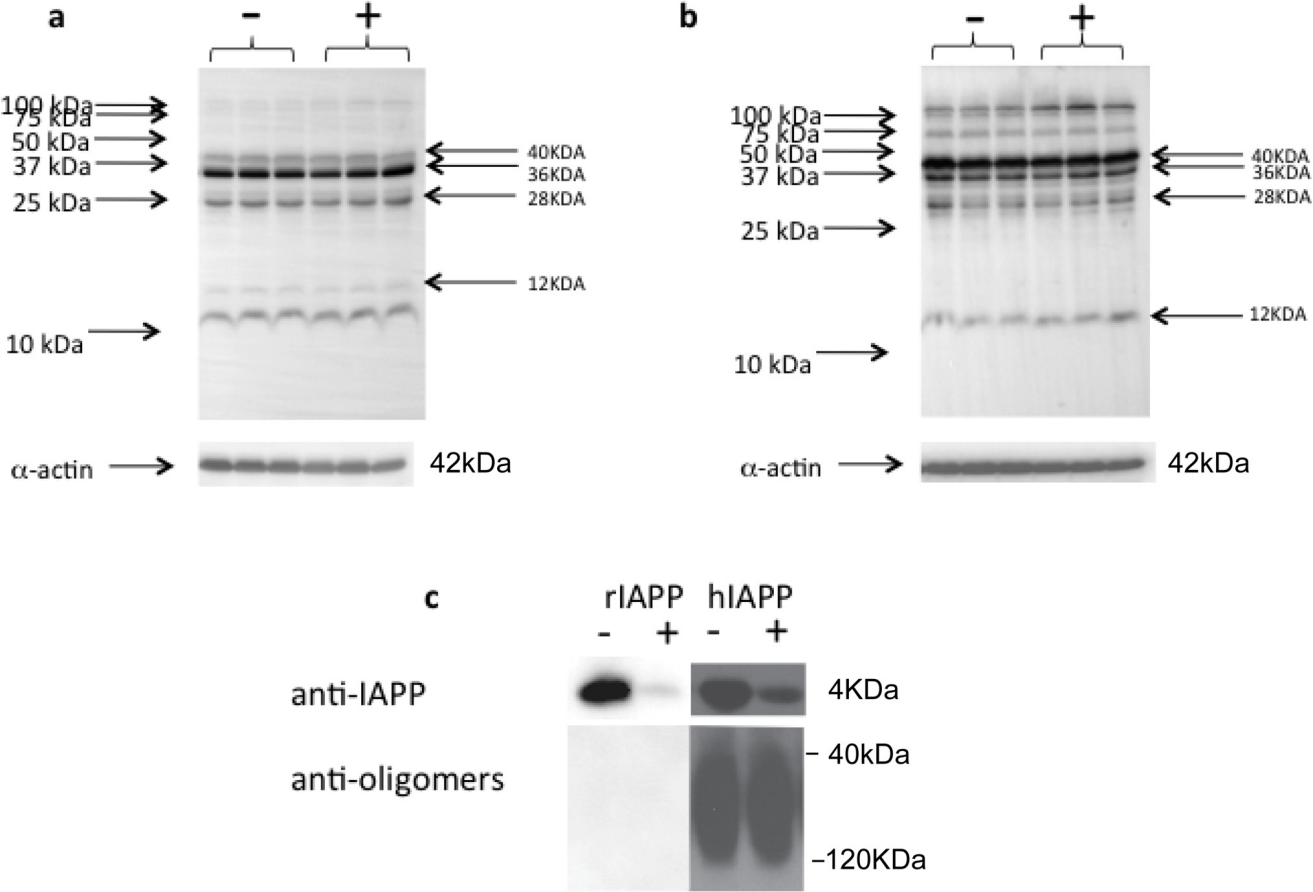
Pitrilysin does not degrade oligomeric hIAPP in vitro. a. Oligomers of hIAPP were generated from hIAPP incubated with rat cardiac myocytes and then incubated with pitrilysin (400 nM) at 37°C for 1 hr. Reactions were analyzed by Western blot analysis using mouse monoclonal anti-human amylin antibody E-5. b. Western blot of hIAPP oligomers generated from HIP rat cardiac myocytes incubated with pitrilysin. c. hIAPP were preincubated at 37°C to induce oligomer formation and then incubated with or without 40 nM pitrilysin at 37°C for 1hr. Reactions were analyzed by Western blots using rabbit anti-IAPP that recognizes monomeric IAPP or rabbit anti-oligomer antibody A11. rIAPP (20 μM) was treated at the same condition as a negative control.

Based on the crystal structure of pitrilysin [33] we would not expect the enzyme to degrade IAPP oligomers since they could not easy fit into the enzyme active site. This was confirmed as shown in **Fig 2,** by the inability of pitrilysin to reduce the amount of hIAPP oligomers of 12 kDa, 28 kDa, 36 kDa, or 40 kDa. These likely correspond to hIAPP trimers, heptamers, nonomers, and decamers, respectively. The same results were obtained using a different antibody rabbit polyclonal, rabbit anti-IAPP T4157. Pitrilysin is also not able to degrade large hIAPP large oligomers (**Fig 2C**). It is worth noting that the amount of pitrilysin used to test for the degradation of hIAPP oligomers would have completely degraded monomeric hIAPP under the reaction conditions employed. As expected rIAPP did not form large oligomers under our experimental conditions. Thus pitrilysin degrades monomeric, but not oligomeric IAPP.

To identify the pitrilysin dependent peptide cleavage sites in hIAPP, reaction products were first separated by HPLC (**Fig 1A**), manually collected and subjected to mass spectral analysis. The identified degradation products are listed in **S1 Table** while a schematic representation of the cleavage sites is shown in **Fig 1C**. Initial cleavage of hIAPP by pitrilysin (thick arrows, **Fig 1C**) occurs between Phe^15^-Leu^16^, Gly^24^-Ala^25^ and Ala^25^-Ile^26^. Products were subsequently cleaved at Arg^11^-Leu^12^, Leu^12^-Ala^13^, Ala^13^-Asn^14^ and Asn^14^-Phe^15^ (thin arrows, **Fig 1C**)_._ The degradation of hIAPP by pitrilysin resulted in the production of several 9–15 amino acid fragments with the most abundant being the carboxy-terminal peptide 25-37-amide. Two of the major cleavage sites (Gly^24^-Ala^25^ and Ala^25^-Ile^26^) are within the GAILS sequence believed to be important for hIAPP aggregation and amyloidogenesis. Since five of the cleavage products were clustered we assayed for carboxypeptidase activity in the pitrilysin preparation with hippuryl-L-phenylalanine as a general carboxypeptidase substrate [22, 34]. No such activity was detected.

### Comparison of the kinetic properties of pitrilysin to other IAPP degrading peptidases

Having established IAPP as a substrate of pitrilysin, the kinetic properties of the reaction were determined. The rate of hIAPP hydrolysis was determined by measuring the disappearance of the substrate by HPLC, while *k*_cat_ was determined based on the linear initial rate of hIAPP hydrolysis at a saturating hIAPP concentration (20 μM) using a molecular mass of 117 kDa for pitrilysin. The K_M_ for hIAPP was determined by using it as an alternate substrate inhibitor of the hydrolysis of the fluorogenic peptide Abz-GGYRRGQ-EDDnp [23]. The k_cat_ and K_M_ values of pitrilysin with hIAPP are 13.5 ± 0.1 min^-1^ and 0.38 ± 0.01 μM, respectively (**Table 1**). Since pitrilysin cleaves amyloid β peptide _1–40_ (Aβ_1–40_) [20, 35], we compared the kinetics with Aβ_1–40_ to that of hIAPP and found that pitrilysin has a ~4 to 4.5 times higher cleavage rate and higher affinity for hIAPP compared to Aβ_1–40_ (the k_cat_ and K_M_ value of pitrilysin with Aβ_1–40_ is 3.3 ± 0.1 min^-1^ and 1.7 ± 0.1 μM, respectively [23]. Thus the catalytic efficiency of pitrilysin towards hIAPP is 18 times higher than towards Aβ_1–40_ (k_cat_/K_M_ = 35.5 and 1.94 min^-1^μM^-1^ for hIAPP and Aβ_1–40_, respectively) suggesting that hIAPP may be a physiologically relevant substrate for pitrilysin.

**Table 1:** Comparison of enzymatic characters of pitrilysin and IDE towards different substrates.

Substrate	Enzyme	k_cat_ (min^-1^)	K_M_ (μM)	k_cat_/K_M_ (min^-1^μM^-1^)	Reference
**hIAPP**	**Pitrilysin**	13.5 ± 0.1	0.38 ± 0.01	35.5	
	**IDE**	294 ± 0.4	3.11 ± 0.20	94.5	
**Aβ**_**40**_	**Pitrilysin**	3.3 ± 0.1	1.7 ± 0.1	1.95	[23]
	**IDE**	221 ± 11	0.87 ± 0.10	250	[43]
**Insulin**	**Pitrilysin**	0.2 ± 0.0	54.5 ± 5.5	0.0037	[23]
	**IDE**	0.56 ± 0.01	0.13 ± 0.02	4.3	[44]

The *k*_cat_ value of pitrilysin towards hIAPP was determined based on the rate of hIAPP hydrolysis at a saturating concentration (20μM) and a molecular mass of 117 kDa for pitrilysin.

The K_M_ value of pitrilysin towards hIAPP was determined using hIAPP as an alternate substrate inhibitor of fluorogenic substrate Abz-GGFYRRVGQ-EDDnp hydrolysis as described in Materials and Methods.

Insulin-degrading enzyme (IDE) has been shown to hydrolyze hIAPP [36]. We therefore compared the enzymatic properties of these two enzymes in terms of hIAPP degradation. The k_cat_ and K_M_ values for IDE with hIAPP were determined as above yielding values of 294 ± 0.4 min^-1^ and 3.11 ± 0.20 μM, respectively. Compared to IDE, the catalytic efficiency of pitrilysin towards hIAPP is similar, exhibiting 40% of the catalytic efficiency of IDE (k_at_/K_M_ = 35.5 and 94.5 min^-1^μM^-1^ for pitrilysin and IDE, respectively). Although IDE is a major insulin degrading enzyme *in vivo*, pitrilysin was shown to be very inefficient in degrading insulin [23] (**Table 1**). Thus relative to IDE, pitrilysin is a more specific IAPP degrading enzyme.

### Pitrilysin expression in pancreatic islets

**Fig 3:**
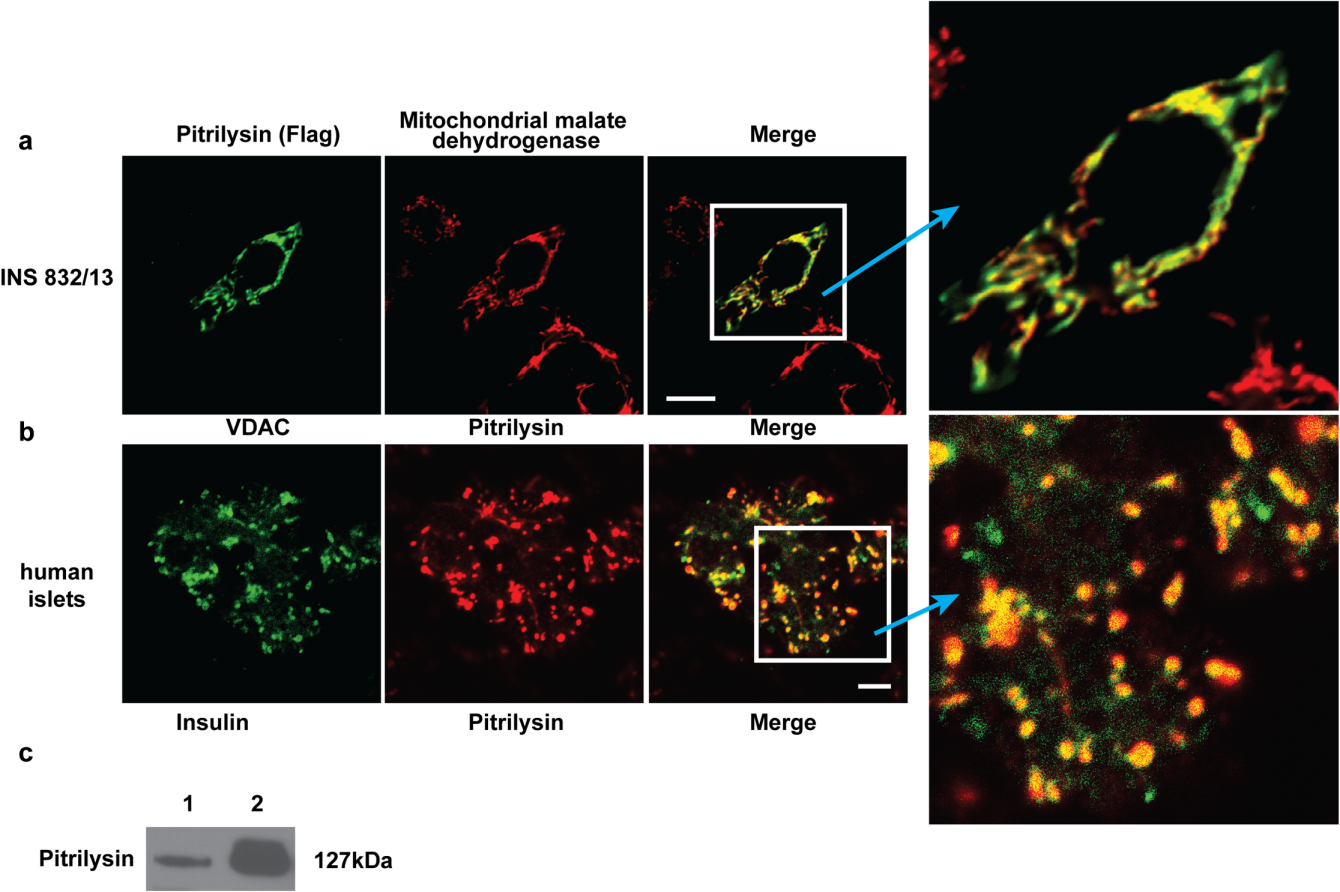
Pitrilysin expression in pancreatic beta-cells. **a.** INS 832/13 cells were transfected with pcDNA3.1-pitrilysin-flag. Forty-eight hrs. after transfection, cells were fixed and stained for flag tagged pitrilysin and mitochondrial MDH. **b.** The mitochondrial location of pitrilysin in human islets evidenced by colocalization of pitrilysin with the mitochondrial marker, voltage-dependent anion channel. **c.** Western blot analysis of pitrilysin in extracts of human islets and subjected to Western blot analysis with polyclonal rabbit anti-pitrilysin antibody. Lane 1: human islet lysate (10 μg); Lane 2: 60 ng recombinant pitrilysin.

Pitrilysin has been shown to be located within the mitochondrial matrix [22,25]. We confirmed the mitochondrial location of pitrilysin by demonstrating its colocalization with mMDH by overexpression of pitrilysin in insulinoma cells INS 832/13 (**Fig 3A**). We also demonstrated a mitochondrial location of pitrilysin in human islets by its colocalization with the mitochondrial voltage-dependent anion channel 1 (**Fig 3B**). Western blot analysis of human islet extracts also showed the presence of pitrilysin (**Fig 3C**).

### Knockdown of pitrilysin in INS cells increases hIAPP-induced apoptosis

**Fig 4:**
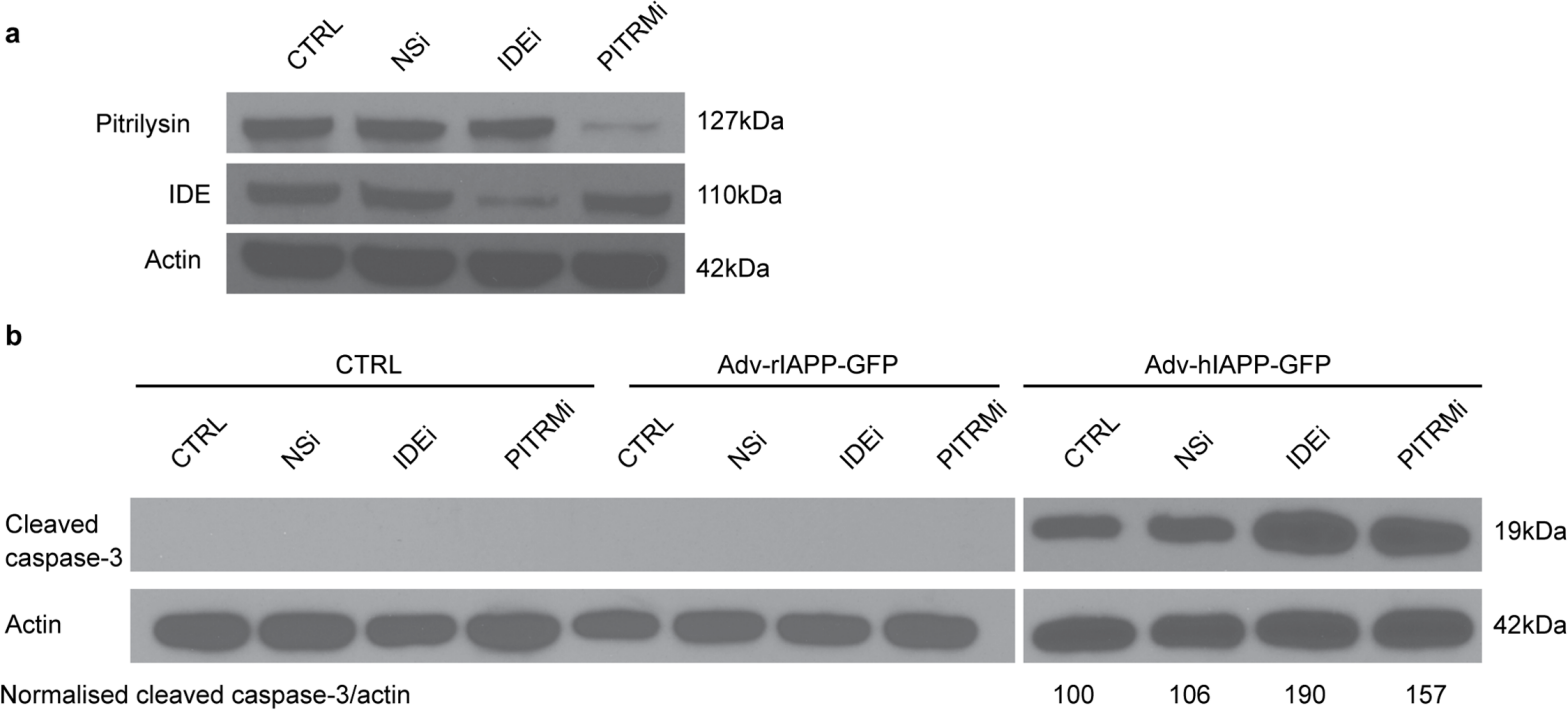
Knockdown of pitrilysin in INS cells exacerbates hIAPP-induced apoptosis. Stably transfected INS 832/13 cells expressing an shRNA specific to rat pitrilysin (PITRMi), rat IDE (IDEi) or non-silencing control shRNA (NSi) were selected using 10 μg/ml puromycin. Stable cells were then transduced with Adv-prepro-hIAPP-GFP (MOI = 100) and apoptosis determined by Western blot analysis of cleaved caspase-3. Band densities were analyzed using NIH Image J software. The ratios of cleaved caspase-3 to actin intensities are indicated (n = 3). **a.** Pitrilysin in INS832/13 cells is reduced ~95% by lenti-PITRMi. **b.** An approximate 60% increase (162.1 ± 13.2% of control, p<0.01) in apoptosis induced by hIAPP in pitrilysin shRNA treated cells compared to the lenti-NSi control or parental INS 832/13 cells. shRNA treatment itself did not induce apoptosis.

To determine whether endogenous pitrilysin regulates IAPP in cells we measured the effect of pitrilysin on preventing hIAPP induced apoptosis. We used RNA interference (RNAi) to decrease pitrilysin and then measured apoptosis produced by hIAPP. We introduced recombinant hIAPP through an adenovirus vector [29] since the endogenous rIAPP does not oligomerize and thus does not induce apoptosis. Cells were transduced with an shRNA specific to rat pitrilysin (PITRMi) and selected with puromycin. Since inhibition of IDE also accelerates hIAPP amyloid formation in insulinoma cells [36, 37], we used an shRNA specific to rat IDE (IDEi) as a positive control and a nonsilencing shRNA (NSi) as a negative control. **Fig 4A** shows that in INS832/13 cells pitrilysin levels were reduced ~95% by the shRNA, while IDE levels were reduced by 80%. Cells were then transduced with adenovirus Adv- prepro-hIAPP-GFP or Adv-prepro-rIAPP-GFP as a negative control, and apoptosis was evaluated by measuring the cleavage of caspase-3.

When the pitrilysin-depleted cells expressed Adv-prepro-hIAPP-GFP, apoptosis significantly increased relative to naïve cells, **Fig 4B**, but not in control cells. This finding confirms hIAPP induced apoptosis. IDE knockdown (IDEi) also increased hIAPP-induced apoptosis as expected. The nonsilencing control shRNA (NSi) did not affect hIAPP-induced apoptosis (**Fig 4B**). Apoptosis was not induced by knockdown of pitrilysin or IDE in the absence of hIAPP expression, nor by treatment with Adv-propro-rIAPP-GFP. These results strongly support a role for pitrilysin in the degradation of hIAPP *in vivo*.

### Overexpression of pitrilysin protects INS cells from hIAPP-induced apoptosis

**Fig 5:**
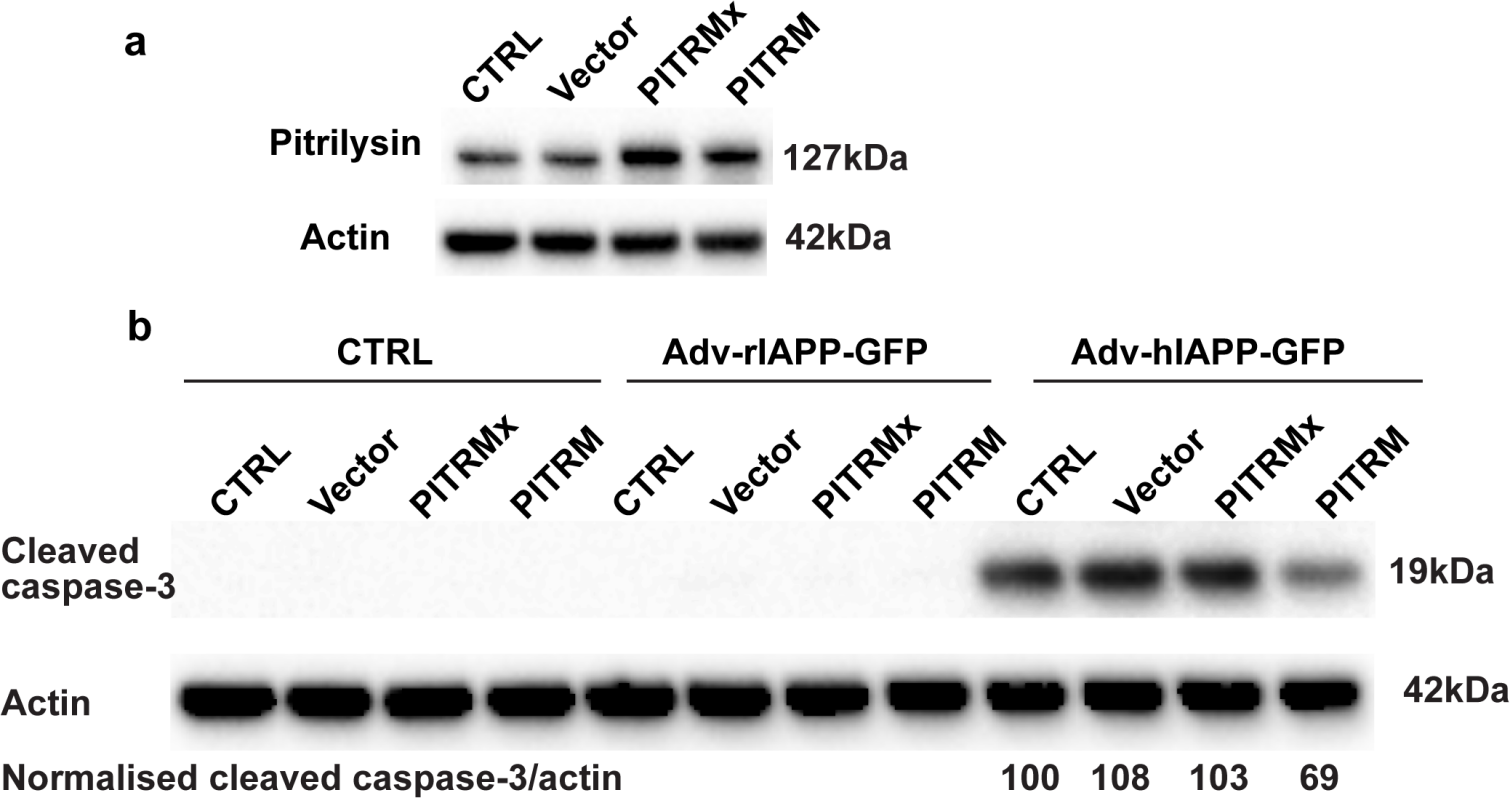
Overexpression of pitrilysin protects INS cells from hIAPP-induced toxicity. INS 832/13 cells were transduced with lentivirus carrying human pitrilysin (PITRM), an inactive mutant (PITRMx), or empty lentiviral vector. Stable cell lines were isolated and then transduced with Adv-prepro-hIAPP-GFP. Apoptosis was determined at 48 hrs. after transduction as in Fig 4 with the ratios of cleaved caspase-3 to actin intensities indicated (n = 3). **a.** Pitrilysin protein levels were increased by 80% (PITRMx) and by 60% (PITRM) in the stable cell lines. **b.** An ~30% decrease (68.7 ± 10.3% of control, p<0.05) in apoptosis induced by hIAPP was observed in pitrilysin overexpressing cells compared to empty lentiviral vector control, inactive pitrilysin (PITRMx) overexpressing cells, or parental INS 832/13 cells. Treatment with Adv-prepro-rIAPP-GFP did not cause apoptosis.

To further examine the role of pitrilysin in regulating IAPP, we used lentivirus carrying human pitrilysin to overexpress the enzyme. Lentivirus carrying an inactive E107Q mutant pitrilysin (PITRMx) and empty lentiviral vector were used as negative controls. **Fig 5A** shows that lentivirus transformation increased pitrilysin levels by 80% for PITRMx and 60% for PITRM. The cells with increased active pitrilysin were more resistant to Adv-prepro-hIAPP-GFP-induced apoptosis compared to naïve cells, cells treated with empty lentiviral vector, as well as cells treated with inactive pitrilysin (**Fig 5B**). As an additional control, cells treated with Adv-prepro-ratIAPP-GFP did not exhibit significant apoptosis.

### Intramitochondrial localization of IAPP in pancreatic beta-cells

Pitrilysin is known to be located within the mitochondrial matrix of cells [20, 23], and no studies have reported its location outside the mitochondrion. Therefore since pitrilysin is a mitochondrial protein and since pitrilysin protects against IAPP induced apoptosis, we questioned whether there is a pool of IAPP inside mitochondria. This is the simplest explanation that could account for pitrilysin’s action on preventing hIAPP induced apoptosis.

**Fig 6:**
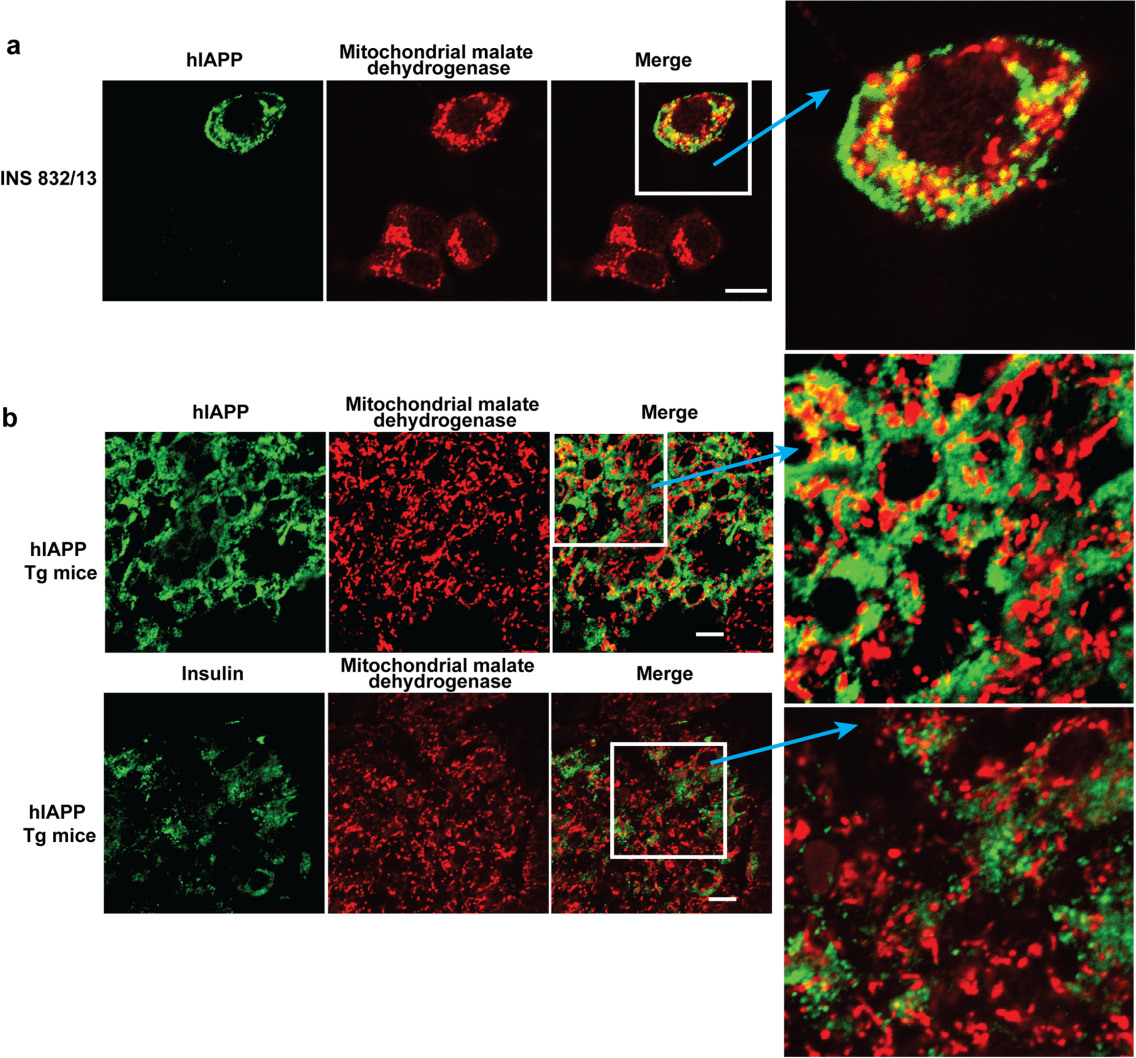
Mitochondrial localization of hIAPP. **a.** INS 832/13 cells were transfected with pCMV6-XL5-prepro-h*IAPP*. Forty-eight hrs. after transfection, cells were fixed and stained for hIAPP (mouse anti-hIAPP) and mitochondrial malate dehydrogenase. **b.** hIAPP/mMDH and insulin/mMDH immunocytochemistry performed on paraffin sections of pancreas from hIAPP transgenic mice. Staining was visualized with FITC conjugated goat anti-mouse and cy3 conjugated goat anti-rabbit secondary antibodies. Confocal images were taken as described in Methods and merged with Adobe Photoshop software. Scale bar: 10μm. Yellow color denotes colocalization.

To determine whether IAPP accumulates in the mitochondria of pancreatic beta-cells, the pancreatic beta-cell line INS 832/13 [28] was transfected with plasmid pCMV6-XL5-prepro-hIAPP producing hIAPP. Using double fluorescence staining, significant amounts of hIAPP appeared to colocalize with the mMDH (**Fig 6A**). Similarly, the colocalization of hIAPP with mMDH was also indicated in pancreas sections of hIAPP transgenic mice, although the resolution of the images were insufficient to distinguish the sub-organelle localization. In this case the immunofluorescence corresponding to mitochondrial hIAPP was ~9% of total hIAPP (**Fig 6B, upper panel**). In contrast and as a control, considerably less insulin appeared colocalized with mMDH (~2% of total insulin immunoreactivity) representing background staining (**Fig 6, lower panel**).

**Fig 7:**
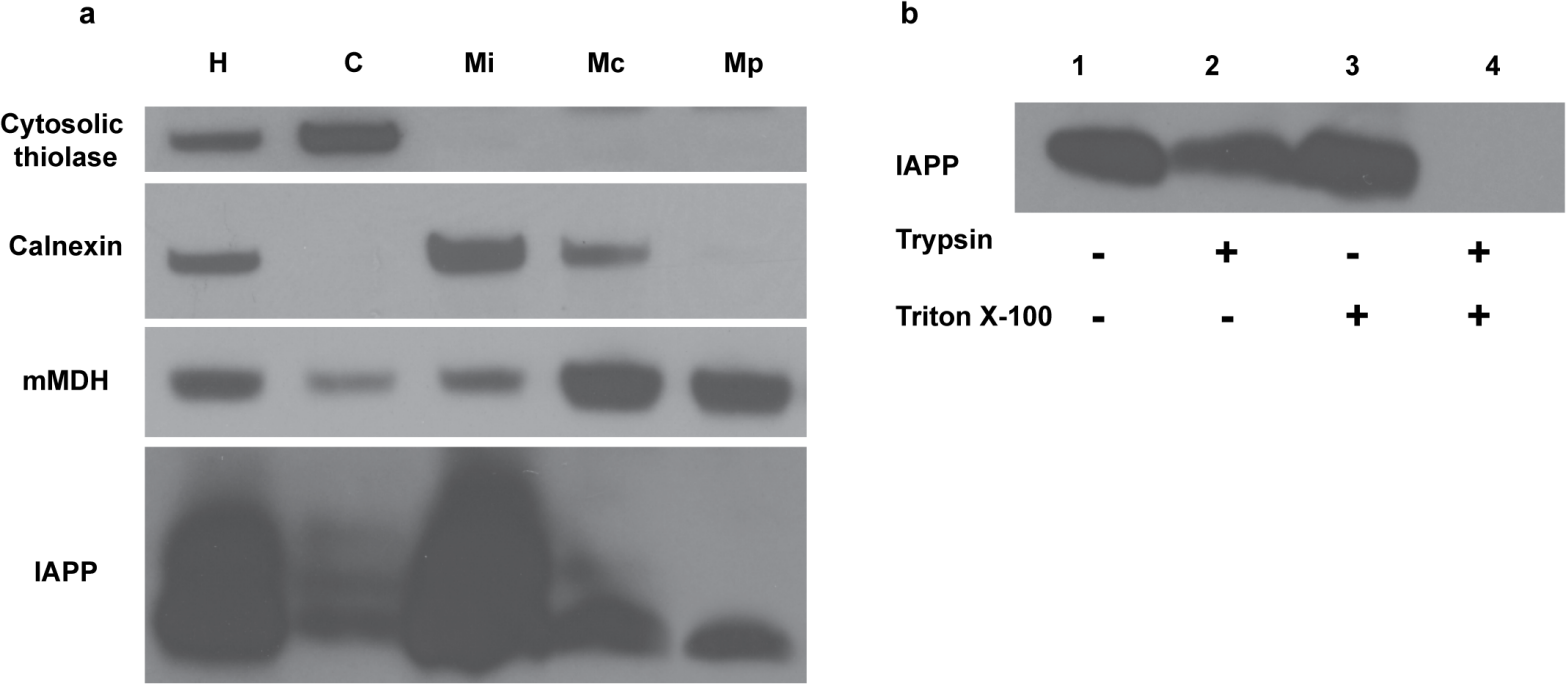
Detection of IAPP in the mitochondrial fraction from INS cells. **a.** Protein components of subcellular fractions prepared from INS cells were immunoblotted with anti-IAPP and antibodies to a cytosolic marker (cytosolic thiolase), an ER marker (calnexin), and a mitochondrial markers (mitochondrial malate dehydrogenase, mMDH). H: homogenate; C: cytosol; Mi: Microsomal fraction; Mc: crude mitochondrial fraction; Mp: purified mitochondrial fraction. **b.** Purified mitochondria from INS cells were incubated with trypsin (10 μg/ml) in the presence or absence of Triton X-100 (0.01%) at 37°C for 30min. The reactions were stopped with 50 μg/ml of trypsin inhibitor and subjected to SDS-PAGE and Western blot analysis. IAPP in mitochondria is insensitive to trypsin without prior Triton X-100 permeabilization suggesting an intramitochondrial location of IAPP.

To provide additional evidence for a mitochondrial location of a pool of IAPP in pancreatic beta-cells and eliminate any confounding effects of hIAPP oligomerization / assemblies on its subcellular location, we examined the localization of endogenous rat IAPP in INS 832/13 cells employing cell fractionation and Western blot analysis [32]. Although hIAPP differs from rIAPP in that the latter does not readily oligomerize, monomeric rIAPP should be similar to monomeric hIAPP in terms of its subcellular localization. As expected the majority of the detectable intracellular rIAPP localized to the microsomal fraction (Mi), mainly endoplasmic reticulum (ER), however a fraction of rIAPP was found to be localized to mitochondria (**Fig 7A**). Although the majority of cellular IAPP appears to be located in the ER, the importance of mitochondrial IAPP cannot be underestimated since as described below mitochondrial pitrilysin likely keeps its steady-state concentration low under normal conditions. Under pathological conditions, the mitochondrial IAPP concentration could increase and be an important factor in inducing mitochondrial dysfunction, similar to the role of mitochondrial amyloid β-peptide in Alzheimer’s disease [38]. Importantly we were able to demonstrate that this mitochondrial rIAPP is inside the mitochondria since it is protected from trypsin cleavage without prior mitochondrial membrane permeabilization (**Fig 7B**). Thus these data indicate a previously unidentified mitochondrial pool of IAPP, which is analogous to the pool of Aβ localized inside mitochondria in Alzheimer’s disease [16, 17].

## Discussion

Pitrilysin was initially described as a metallopeptidase cleaving the opioid peptide leumorphin [39] and later shown to cleave Aβ [20, 35]. We now show that pitrilysin is able to efficiently cleave hIAPP. The overall degradation of hIAPP by pitrilysin results in the production of a number of small peptides with the most abundant fragment being the COOH-terminal peptide hIAPP^25–37^-amide. Cleavage of hIAPP by pitrilysin produces non-toxic products, likely due to cleavages in the region of hIAPP known to be important for its oligomerization. Pitrilysin is ~20 times more efficient in cleaving hIAPP than Aβ. Thus, if pitrilysin is able to degrade Aβ *in vivo* [20], it should also be able to degrade hIAPP *in vivo*. Our demonstration of pitrilysin in human islets suggests pitrilysin plays a role in regulating IAPP levels in humans.

Given that knockdown of pitrilysin increased hIAPP-induced apoptosis and that overexpression of pitrilysin protects against hIAPP-induced apoptosis, this data strongly suggest that pitrilysin contributes to the regulation of cellular IAPP levels. The fact that pitrilysin is known to be a mitochondrial enzyme and the data presented here that suggests co-localization of a fraction of IAPP with the mitochondrial marker mitochondrial malate dehydrogenase, these findings raise the intriguing possibility that there exists an intramitochondrial pool of IAPP which contributes to IAPP induced beta-cell death. Since pitrilysin degrades monomeric, but not oligomeric IAPP, this putative mitochondrial pool of hIAPP must contain monomeric IAPP. However, this mitochondrial hIAPP could aggregate to form toxic oligomers within the mitochondrion. How monomeric IAPP is transported into mitochondria is currently unclear.

IAPP is synthesized in the ER as a precursor protein, which is then processed to its mature form and secreted into the extracellular space [40]. hIAPP can be internalized by cells when exogenously applied [41, 42], however extracellular monomeric IAPP is taken up by endocytosis and trafficked into late endosomes or lysosomes from which it is cleared [41]. Extracellular aggregates of hIAPP take on cell penetrating protein properties and can be translocated across the cell membrane into the cytoplasm, where they can interact with the mitochondrial outer membrane and induce mitochondrial dysfunction [41]. In addition, toxic oligomers of hIAPP can be formed intracellularly within the secretory pathway where they disrupt membranes and are released into the cytoplasm [7]. These secretory pathway derived oligomers can bind to and disrupt the outer mitochondrial membranes producing mitochondrial dysfunction and apoptosis. However, none of these IAPP pools would be substrates for pitrilysin, which resides inside the mitochondrion. It is interesting to note that in the published EM micrographs of Gurlo *et al*. [7], one can see anti-IAPP staining in islet mitochondria, consistent with intramitochondrial IAPP.

## Supporting Information

S1 Fig Purity of recombinant pitrilysin analyzed by SDS-PAGE. Recombinant pitrilysin was purified as described in the Methods section and analyzed by SDS-PAGE on a 10% polyacrylamide gel stained with Coomassie blue. The purity of recombinant pitrilysin is greater than 97%.
(TIF)

S1 Table hIAPP cleavage fragments identified by Mass spectral analysis. 20μM hIAPP was incubated with 40 nM recombinant pitrilysin at 37°C and the degradation of hIAPP was analyzed by HPLC. Peaks were collected manually and subjected to mass spectral analysis for identification. Peak designations are shown in Fig 1A.
(DOCX)
