# Supplementary figures and images for: The Mitochondrial Peptidase Pitrilysin Degrades Islet Amyloid Polypeptide in Beta-Cells
Source: PLoS One. 2015 Jul 20;10(7):e0133263. doi: 10.1371/journal.pone.0133263 (PMC4507941; doi:10.1371/journal.pone.0133263)

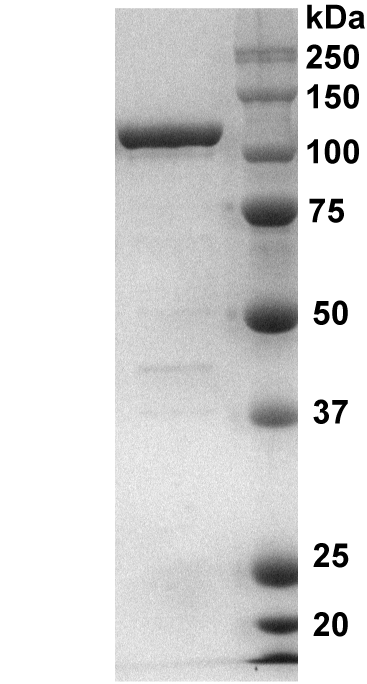

Supplement: S1 Fig — Recombinant pitrilysin was purified as described in the Methods section and analyzed by SDS-PAGE on a 10% polyacrylamide gel stained with Coomassie blue. The purity of recombinant pitrilysin is greater than 97%. (TIF) [file pone.0133263.s001.tif]
