# Supplementary material for: The Mitochondrial Peptidase Pitrilysin Degrades Islet Amyloid Polypeptide in Beta-Cells
Source: PLoS One. 2015 Jul 20;10(7):e0133263. doi: 10.1371/journal.pone.0133263 (PMC4507941; doi:10.1371/journal.pone.0133263)
Supplement: S1 Table — 20μM hIAPP was incubated with 40 nM recombinant pitrilysin at 37°C and the degradation of hIAPP was analyzed by HPLC. Peaks were collected manually and subjected to mass spectral analysis for identification. Peak designations are shown in Fig 1A. (DOCX) [file pone.0133263.s002.docx]

**Supporting Information**

**Table S1. hIAPP cleavage fragments identified by Mass spectral analysis.**

20μmol/l hIAPP was incubated with 40 nmol/l recombinant pitrilysin at 37°C and the degradation of hIAPP was analyzed by HPLC. Peaks were collected manually and subjected to mass spectral analysis for identification. Peak designations are shown in Fig. 6a.

| **Peak** | **Fragment identified** | **Observed Mass (MH^+^)** | **Expected Mass(MH^+^)** |
| --- | --- | --- | --- |
| a | LVHSSNNFG | 974.45 | 974.46 |
| b | LVHSSNNFGA | 1045.50 | 1045.49 |
| c | KCNTATCATQRLAN** | 1492.69 | 1492.70 |
| d | ILSSTNVGSNTY-NH2 | 1254.63 | 1254.61 |
| e | AILSSTNVGSNTY-NH2 | 1325.68 | 1325.65 |
| f | FLVHSSNNFG | 1121.57 | 1121.53 |
| g | FLVHSSNNFGA | 1192.57 | 1192.57 |
| h | NFLVHSSNNFG | 1235.57 | 1235.57 |
| i | KCNTATCATQRLANF** | 1639.76 | 1639.78 |
| j | LANFLVHSSNNFG | 1419.68 | 1419.69 |
| k | LVHSSNNFGAILSSTNVGSNTY-NH2 | 2281.07 | 2281.10 |
| l | KCNTATCATQRLANFLVHSSNNFG** | 2595.32 | 2595.22 |
| m | KCNTATCATQRLANFLVHSSNNFGA** | 2666.31 | 2666.26 |
